# Dr. Leroy Dwight Farnham

**Published:** 1916-03

**Authors:** 


**Dr. Leroy Dwight Farnham,** P. & S. (Columbia) 1878, died at Binghamton Jan. 3.

